# Eosinophilic pleural effusion due to lung cancer has a better prognosis than non-eosinophilic malignant pleural effusion

**DOI:** 10.1007/s00262-021-02994-5

**Published:** 2021-06-25

**Authors:** Eiji Takeuchi, Yoshio Okano, Hisanori Machida, Katsuhiro Atagi, Yoshihiro Kondou, Naoki Kadota, Nobuo Hatakeyama, Keishi Naruse, Tsutomu Shinohara

**Affiliations:** 1 Department of Clinical Investigation, National Hospital Organization Kochi Hospital, 1-2-25 Asakuranishimachi, Kochi-city, Kochi 780-8507 Japan; 2 Department of Respiratory Medicine, National Hospital Organization Kochi Hospital, 1-2-25 Asakuranishimachi, Kochi-city, Kochi 780-8507 Japan; 3 Department of Pathology, National Hospital Organization Kochi Hospital, 1-2-25 Asakuranishimachi, Kochi-city, Kochi 780-8507 Japan; 4 Department of Community Medicine for Respirology, Graduate School of Biomedical Sciences, Tokushima University, 3-18-15 Kuramoto-cho, Tokushima, 770-8503 Japan

**Keywords:** Lung cancer, Malignant pleural effusion, Eosinophilic pleural effusion, Survival, Better prognosis

## Abstract

**Objective:**

Tumor-related eosinophilia may have extended survival benefits for some cancer patients. However, there has been no report on the prognosis difference between eosinophilic pleural effusion (EPE) and non-EPE in lung cancer patients. Our study aimed to investigate the prognosis difference between EPE and non-EPE due to lung cancer.

**Patients and methods:**

We retrospectively reviewed patients diagnosed with lung cancer who presented with malignant pleural effusion (MPE) between May 2007 and September 2020 at the National Hospital Organization Kochi Hospital. EPE is defined as pleural fluid with a nucleated cell count containing 10% or more eosinophils.

**Results:**

A total of 152 patients were included: 89 were male (59%). The median age was 74.4 years (range 37–101), and all patients were pathologically shown to have MPE. Most patients (140; 92%) had an Eastern Cooperative Oncology Group (ECOG) Performance Status (PS) of 0/1. Twenty patients had EPE. The median overall survival (OS) of all 152 lung cancer patients with MPE was 298 days. The median OS of the patients with EPE was 766 days, and the median OS of the patients with non-EPE was 252 days. Kaplan–Meier univariate analysis showed that lung cancer patients with EPE had a significantly better prognosis than patients with non-EPE (P < 0.05). Cox proportional regression analysis showed that EPE, ECOG PS, sex, and the neutrophil-to-lymphocyte ratio in the serum (sNLR) may be independent prognostic factors affecting survival in patients with MPE.

**Conclusion:**

Lung cancer patients with EPE have a better prognosis than those with non-EPE.

## Introduction

Eosinophilic pleural effusion (EPE) is defined as pleural fluid with a nucleated cell count containing 10% or more eosinophils [1]. It is estimated that approximately 10% of exudative pleural effusions are eosinophilic [1]. Causes of EPE include pleuritis, trauma (*e.g*., pneumothorax, hemothorax, thoracic surgery), and malignancies. According to a meta-analysis of 687 cases of EPE, the most common cause was malignancy (26%), followed by idiopathic (25%) and pneumonia (13%) [2].

On the other hand, it has been shown that tumor-related eosinophilia may have extended survival benefits for some cancer patients [3–5]. In recent years, it has been demonstrated that peripheral blood eosinophils before administration are a potential predictive marker for a beneficial clinical response in cancer immunotherapy, particularly immune checkpoint inhibitors [6–11]. This finding suggested malignant pleural effusion (MPE) is an essential tool for investigating the tumor microenvironment (TME). In a previous prospective study, the survival of patients with EPE was better than that of patients with non-EPE [1]. However, there has been no study on the prognosis difference between EPE and non-EPE in lung cancer patients. Therefore, in this study, we investigated the prognosis difference between EPE and non-EPE due to lung cancer.

## Materials and methods

### Patients

We retrospectively reviewed all patients diagnosed with lung cancer who presented with malignant pleural effusion between May 2007 and September 2020 at the National Hospital Organization Kochi Hospital. Only patients with malignant cells confirmed in the pleural fluid or pleural biopsy were included to maintain study quality. We performed conventional cytology examination or histological analyses independently to identify malignant cells in the effusion fluid or pleural biopsy tissue. For conventional cytologic examination, 5 ~ 10 mL of effusion fluid obtained by diagnostic thoracentesis was centrifuged at 2500 rpm for 10 min. We prepared a minimum of two thin smears from the sediment. According to the hospital pathology laboratory's standard protocol, one smear was air-dried and stained with Leishman-Giemsa stain. The other smear was immediately fixed in 95% alcohol and stained with Papanicolaou stain. For the histological analysis, tissue specimens obtained during the pleural biopsy were processed after formalin fixation, and the sections were stained with hematoxylin–eosin dye. The institutional review board of the National Hospital Organization Kochi Hospital approved the study protocol. Informed consent was waived because of the retrospective nature of the study.

## Statistical analysis

Categorical and continuous variables are summarized using descriptive statistics. The independent-samples *t* test was used to test for differences between continuous variables. The Pearson's chi-squared test and Fisher’s exact test were used to test for associations between categorical variables. Overall survival (OS) was evaluated as the period from the day when pleural effusion was collected to the day of death from any cause using the Kaplan–Meier method. The log-rank test was used to compare survival curves. A Cox proportional hazards model was used to estimate the hazard ratio (for eosino ≥ 10% compared with eosino < 10%) with a 95% confidence interval (CI). All statistical analyses were performed using EZR (developed in 2012 by Y. Kanda, Saitama Medical Center, Jichi Medical University), a graphical user interface in R (version 3.6.3, R Foundation for Statistical Computing, Vienna, Austria) or SPSS statistics version 27.0 (IBM, Armonk, USA). P-values are presented without adjusting for multiple comparisons in an exploratory manner.

## Results

### Patient characteristics

A total of 152 lung cancer patients with MPE were included in the study. Among the 152 patients, 20 patients had EPE. The clinical characteristics of the enrolled patients are summarized in Table 1. Among the 152 patients, the mean age at diagnosis was 74.4 years (range 37–101), 89 patients (59%) were male, and 67 patients (44%) were former or current smokers. The majority of patients had an Eastern Cooperative Oncology Group (ECOG) Performance Status (PS) of 0–1 (92%) and exhibited adenocarcinoma histology (82%). Twenty-seven patients were harboring epidermal growth factor receptor (EGFR) mutations and received EGFR-tyrosine kinase inhibitors. Fifty-nine patients (39%) received pleurodesis. Among the patients, 34% and 66% received supportive care only and active treatment, respectively.

**Table 1:** Characteristics of the study population

		Total	Eosino ≥10%	Eosino <10%	p
		n=152	n=20	n=132	
Age	Mean age, years (range)	74.4 (37-101)	71.4 (37-101)	74.8 (45-100)	0.10*
	≥65 (%)	126 (82)	16 (80)	110 (83)	
	<65 (%)	26 (17)	4 (20)	22 (17)	
Sex (%)	Male	89 (59)	11 (55)	78 (59)	0.81**
	Female	63 (41)	9 (45)	54 (41)	
Smoking history (%)	Yes	67 (44)	10 (50)	58 (44)	0.82***
	No	63 (41)	7 (35)	56 (42)	
	Missing	22 (14)	3 (15)	18 (14)	
ECOG PS (%)	0	19 (12)	3 (15)	16 (12)	0.76***
	1	121 (80)	17 (85)	104 (79)	
	2	7 (5)	0 (0)	7 (5)	
	3	2 (1)	0 (0)	2 (2)	
	4	3(2)	0 (0)	3 (2)	
pLHD	Mean, IU/L	719	538.9	746.5	0.26*
	(range)	(81-13200)	(136-2287)	(81-13200)	
sNLR	Ratio	6.75	7	6.71	0.34*
	(range)	(0.96-66.62)	(1.17-66.62)	(0.96-63.74)	
Histologic type (%)	Adeno	125 (82)	18 (90)	107 (81)	0.36***
	Squamous	6 (4)	1 (5)	5 (4)	
	Small	11 (7)	0 (0)	11 (8)	
	Others	10 (7)	1 (5)	9 (7)	
Driver mutation (%)	EGFR	27 (18)	1 (5)	26 (20)	0.20**
	ALK	1 (1)	1 (5)	0 (0)	0.13**
Pleurodesis (%)	Yes	59 (39)	9 (45)	50 (38)	0.63**
	No	93 (61)	11 (55)	82 (62)	
Treatment (%)	Supportive	52 (34)	6 (30)	46 (35)	1.00**
	Systemic chemotherapy	100 (66)	14 (70)	86 (65)	

ECOG PS, Eastern Cooperative Oncology Group Performance Status; pLHD, pleural fluid lactate dehydrogenase; sNLR, neutrophil-to-lymphocyte ratio in the serum; EGFR, epidermal growth factor receptor; ALK, anaplastic lymphoma kinase

^*^Independent-samples *t* test; ** Fisher’s exact test; *** Chi-squared test

Among the 20 patients with EPE, the mean age at diagnosis was 71.4 years (range 37–101), 11 patients (55%) were male, and 10 patients (50%) were former or current smokers. All patients had an ECOG PS of 0–1, and 18 patients (90%) exhibited adenocarcinoma histology. Only one patient was harboring EGFR mutations and received EGFR-tyrosine kinase inhibitors. Also, only one patient was harboring anaplastic lymphoma kinase (ALK) rearrangement and received ALK-tyrosine kinase inhibitors. Nine patients (45%) received pleurodesis. Among the patients, 30% and 70% received supportive care only and active treatment, respectively.

Among the 132 patients with non-EPE, the mean age at diagnosis was 74.8 years (range 45–100), 78 patients (59%) were male, and 58 patients (44%) were former or current smokers. The majority of patients had an ECOG PS of 0–1 (91%) and exhibited adenocarcinoma histology (81%). Of the 132 patients with non-EPE lung cancer, older patients, small cell carcinoma and EGFR mutations appeared to be slightly more common than EPE. However, there was no significant difference in patient characteristics between lung cancer patients with EPE and non-EPE. Furthermore, there was no bias in the presence or absence of active treatment in both groups.

## OS of all lung cancer patients with MPE

**Fig. 1:**
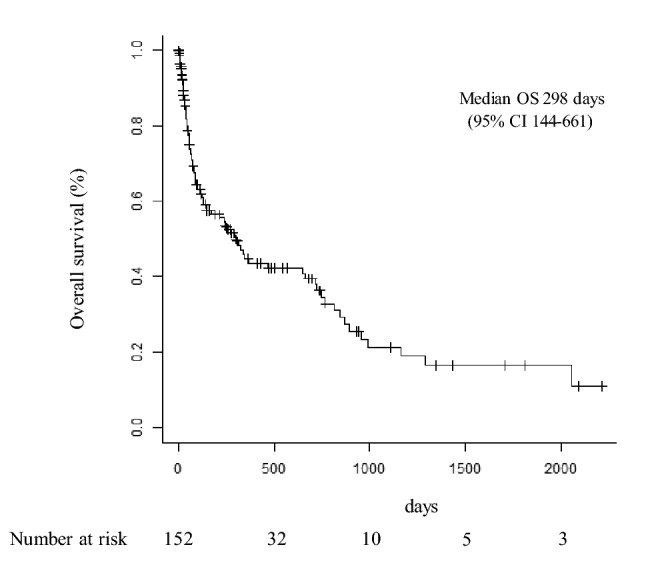
Overall survival (OS) of all 152 lung cancer patients

The OS of all 152 lung cancer patients with MPE was 298 days (95% CI: 144–661) (Fig. 1).

## OS of lung cancer patients with EPE and non-EPE

**Fig. 2:**
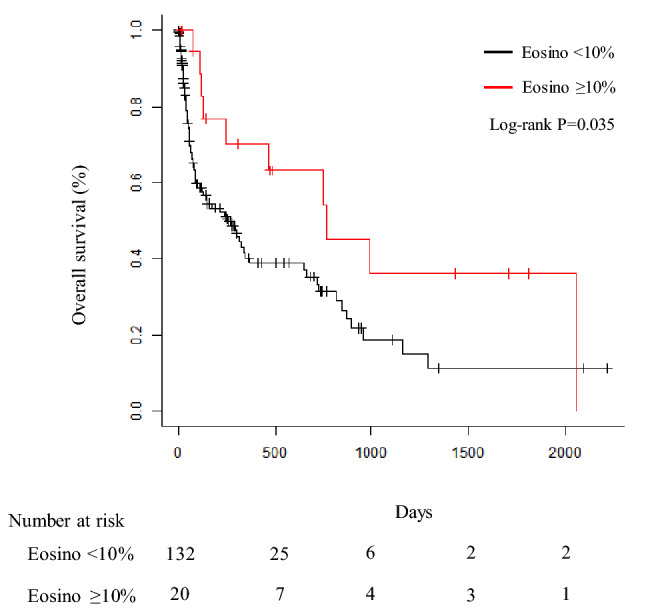
Overall survival of patients with eosinophilic pleural effusion or non-eosinophilic pleural effusion

The OS of lung cancer patients with EPE (n = 20) and non-EPE (n = 132) was 766 days (95% CI: 131-not reached) and 252 days (95% CI: 88–368), respectively (Fig. 2). The OS of lung cancer patients with EPE was significantly longer than that with non-EPE (P = 0.035).

## OS of lung cancer patients with MPE according to the ECOG PS

**Fig. 3:**
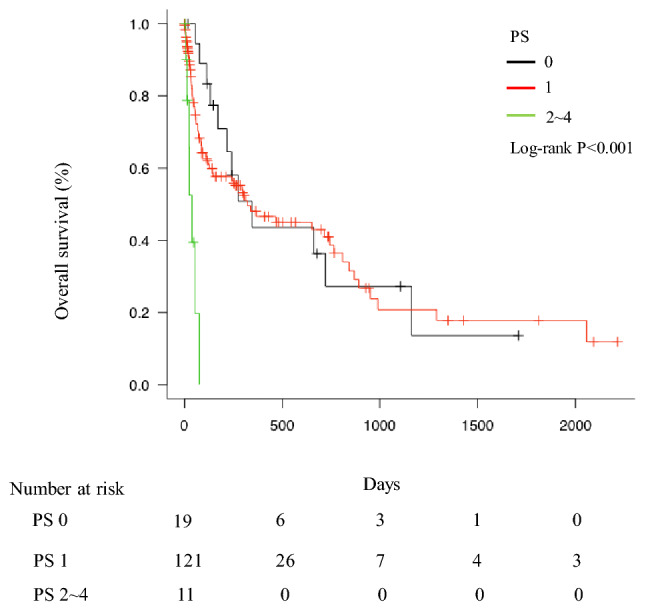
Patient survival according to the ECOG PS

The OS of PS0 (n = 19) lung cancer patients with MPE, PS1 (n = 121), and PS2 ~ 4 (n = 11) were 345 days (95% CI: 171–1162), 323 days (95% CI: 128–774), and 39 days (95% CI: 6-not reached), respectively (Fig. 3). The OS of PS0/1 lung cancer patients with MPE was significantly longer than that of PS2-4 lung cancer patients with MPE (P < 0.001).

## Patient survival according to sex

**Fig. 4:**
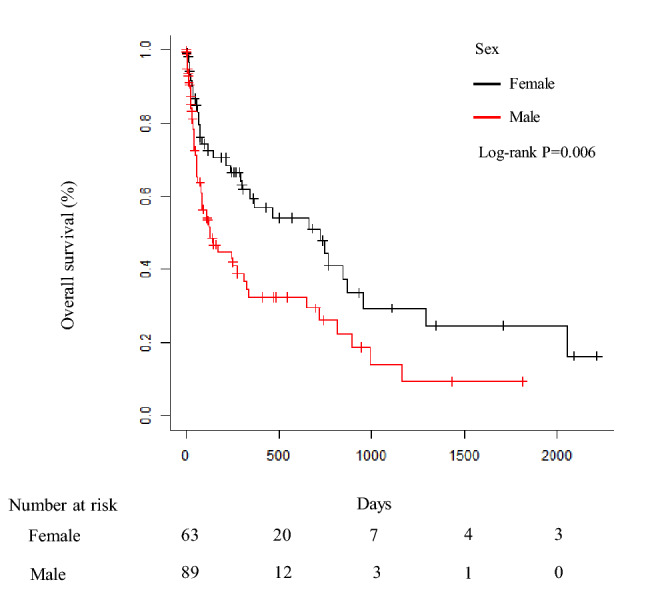
Patient survival according to sex

The OS of female lung cancer patients (n = 63) and of male lung cancer patients (n = 89) was 723 days (95% CI: 293–871) and 131 days (95% CI: 78–311), respectively (Fig. 4). The OS of female lung cancer patients was significantly longer than that of male lung cancer patients (P < 0.006).

## Multivariate analysis

A Cox proportional regression analysis showed that eosino ≥ 10%, ECOG PS, sex, and the neutrophil-to-lymphocyte ratio in the serum (sNLR) may be independent prognostic factors affecting the survival of patients with MPE (Table 2). The histological type was not associated with the prognosis.

**Table 2:** Cox proportional regression analysis of statically significant prognostic factors (by univariate analysis) for the survival of 152 patients

Factors	Hazard ratios	95% CI of HR	P-value
Eosino ≥ 10%	0.45	0.22–0.92	0.02
ECOG PS	4.29	1.08–10.20	< 0.001
Sex	1.72	1.08–2.74	0.02
sNLR	1.07	1.04–1.10	< 0.001
NSCLC	1.76	0.79–3.91	0.16

ECOG PS, Eastern Cooperative Oncology Group Performance Status; sNLR, neutrophil-to-lymphocyte ratio in the serum; NSCLC, non-small-cell lung cancer

## Discussion

Our study demonstrates that lung cancer patients with EPE have a better prognosis than those with non-EPE. To our knowledge, this is the first study to show a better prognosis of malignant EPE in lung cancer patients.

EPE is defined as pleural fluid with a nucleated cell count containing 10% or more eosinophils [1]. It is considered that about 10% of exudative pleural effusion is EPE [1]. Causes of EPE include pleuritis, trauma (*e.g*., pneumothorax, hemothorax, thoracic surgery), and malignancies. In a meta-analysis of 687 cases of EPE, the most common cause was malignant tumors (26%), followed by idiopathic (25%) and pneumonia (13%) [2]. On the other hand, the frequency of EPE (about 2.3–6.8%) was not as high in MPE [12, 13]. However, in EPE, the frequency of malignant tumors was 6–40% [1, 14, 15]. Malignant tumors are found in EPE as often as in non-EPE [1]. Lung cancer is the most common malignant tumor associated with EPE [14, 15]. In a review of 135 EPE cases, 47 were associated with malignancies, 23 of which were associated with lung cancer [15].

Pleural fluid lactate dehydrogenase, histology of the primary tumor, ECOG PS, and sNLR have been reported to be predictors of survival in patients with malignant pleural effusion [16–19]. In our study, a multivariable analysis confirmed that eosino ≥ 10% (P = 0.02), PS (P < 0.001), sex (P = 0.02), and sNLR (P < 0.001) may be independent predictors of OS. In previous reports, female lung cancer patients with MPE had a favorable prognosis [18, 19]. These results are consistent with previous studies. Eighteen females and nine males harbored EGFR mutations, and more female lung cancer patients harbored EGFR mutations than males (P < 0.001). Lung cancer patients harboring EGFR mutations have a good prognosis (results not shown), which may have influenced the prognosis in females. However, there have been no reports of good prognosis for EPE in lung cancer patients. We demonstrate for the first time that lung cancer patients with EPE have a better prognosis than those with non-EPE.

In recent years, the relationship between eosinophils and cancer has attracted attention due to cancer immunotherapies such as immune checkpoint inhibitors. Although the functional role of eosinophils in human cancer is not fully understood, many studies have shown that tumor-related eosinophilia may provide survival benefits to cancer patients [3–5]. In oral squamous epithelial cancer, nasopharyngeal cancer [20], esophageal cancer [21], colorectal cancer [22, 23], lung cancer [24], laryngeal cancer, bladder cancer [25], prostate cancer [26], and penis cancer [27], similar findings have been reported. The prognosis was good when there was eosinophil infiltration into the tissue and eosinophil degranulation in the tumor tissue. Good prognosis with eosinophils is also independent of common prognostic factors (stage, age, sex, drinking history, smoking history, histological grading, angiogenesis, vascular infiltration, and nerve infiltration). Interestingly, this findings becomes significant in the subgroup of patients with poor prognosis [20]. On the other hand, tumor-related tissue eosinophil infiltration is a poor prognostic factor in Hodgkin lymphoma [28]. In a knockout model, tumor-related tissue eosinophil infiltration was a risk factor for oral cancer [29]. Eosinophils have been suggested to play pleiotropic and opposing roles in the TME [30–32].

In humans, peripheral blood eosinophilia often occurs when immunotherapy with interleukin (IL)-2 [33, 34], IL-4 [35], granulocyte macrophage colony-stimulating factor [36], or tumor vaccine is performed [37]. Intrapleural administration of IL-2 is also known to cause significant eosinophilic pleural effusion [38]. In recent years, it has been shown that the number of peripheral blood eosinophils before administration is a valuable marker for predicting the effect when using cancer immunotherapies, especially immune checkpoint inhibitors [6–11]. Eosinophils infiltrate the tumor, and their activation there helps T cells infiltrate the tumor as well [4, 39]. It has also been reported that activated eosinophils promote tumor-specific CD8^+^T cell infiltration and tumor rejection and also prolong survival by improving the TME [39].

Activated innate lymphoid cell populations accumulate in human tumor tissues [40]. It has been suggested that lung group 2 innate lymphoid cells (ILC2), which produce IL-5 or IL-13 in response to IL-25 and IL-33, suppress the lung metastasis of cancer cells [41]. Tissue-specific ILC2 infiltrate pancreatic ductal adenocarcinomas to activate tissue-specific tumor immunity, and increased tissue infiltrates of ILC2 correlate with prolonged survival [42]. Eosinophils and LC2 may be important cells that modulate innate and adaptive immunity. Pleural fluid eosinophilia only has a weak correlation with peripheral blood eosinophilia (results not shown). Since eosinophils in the pleural effusion are not normal cells of the lung or pleural tissue, the development of EPE requires the recruitment of eosinophils from the bone marrow [43]. The mechanism of eosinophil recruitment into the pleural space has not yet been fully elucidated. However, cytokines, chemokines, and adhesion molecules are known to be involved. The involvement of ILC2 has also been suggested [44]. Furthermore, it has been reported that ILC2 is present in human MPE and produces type 2 cytokines such as IL-4, IL-5, and IL-13 [45].

The limitation of this study is that it is a single-center retrospective analysis conducted with heterogeneous data regarding patient cohorts. Therefore, the study results are speculative and not definitive. Furthermore, the observation period is 14 years, and advances in treatment may affect the results. However, in previous reports, talc pleurodesis and active treatment were not factors for better survival in MPE patients [17, 19]. In our study, patients with active treatment have a better prognosis than those with supportive care only (results not shown). However, there was no treatment bias between EPE and non-EPE patients. Additionally, active treatment was excluded from the multivariate analysis variables. Finally, the frequency of EPE was not very high in malignant pleural effusions. Overall, a prospective controlled study with multiple centers is needed to confirm our conclusions.

Although we should consider these limitations when interpreting our study, this is the first study showing a better prognosis of lung cancer patients with EPE. The mechanism of the onset of malignant EPE has not yet been fully elucidated, although it is known that host-tumor cell interactions cause eosinophilic pleural effusion. Furthermore, eosinophils may play an essential role in the modulation of innate and adaptive immunity. In general, the relationship between eosinophils and cancer immunology needs further elucidation.

## Conclusion

In conclusion, we demonstrate that lung cancer patients with EPE have a better prognosis than those with non-EPE. In recent years, the relationship between eosinophils and cancer has attracted attention due to cancer immunotherapy, but further progress in basic and clinical cancer research in this field is required.

## List of symbols

ALK: : Anaplastic lymphoma kinase
CI: : Confidence interval
ECOG: : Eastern Cooperative Oncology Group
EGFR: : Epidermal growth factor receptor
EPE: : Eosinophilic pleural effusion
IL: : Interleukin
ILC2: : Group 2 innate lymphoid cells
MPE: : Malignant pleural effusion
OS: : Overall survival
PS: : Performance Status
sNLR: : Neutrophil-to-lymphocyte ratio in the serum
TME: : Tumor microenvironment

## References

[1] Rubins JB, Rubins HB (1996). Etiology and prognostic significance of eosinophilic pleural effusions. Prospect Study Chest.

[2] Oba Y, Abu-Salah T (2012). The prevalence and diagnostic significance of eosinophilic pleural effusions: a meta-analysis and systematic review. Respiration.

[3] Gatault S, Legrand F, Delbeke M, Loiseau S, Capron M (2012). Involvement of eosinophils in the anti-tumor response. Cancer Immunol Immunother.

[4] Davis BP, Rothenberg ME (2014). Eosinophils and cancer. Cancer. Immunol Res.

[5] Varricchi G, Galdiero MR, Loffredo S, Lucarini V, Marone G, Mattei F, Marone G, Schiavoni G (2018). Eosinophils: The unsung heroes in cancer?. Oncoimmunology.

[6] Heppt MV, Heinzerling L, Kähler KC (2017). Prognostic factors and outcomes in metastatic uveal melanoma treated with programmed cell death-1 or combined PD-1/cytotoxic T-lymphocyte antigen-4 inhibition. Eur J Cancer.

[7] Moreira A, Leisgang W, Schuler G, Heinzerling L (2017). Eosinophilic count as a biomarker for prognosis of melanoma patients and its importance in the response to immunotherapy. Immunotherapy.

[8] Gebhardt C, Sevko A, Jiang H (2015). Myeloid Cells and Related Chronic Inflammatory Factors as Novel Predictive Markers in Melanoma Treatment with Ipilimumab. Clin Cancer Res.

[9] Weide B, Martens A, Hassel JC (2016). Baseline Biomarkers for Outcome of Melanoma Patients Treated with Pembrolizumab. Clin Cancer Res.

[10] Tanizaki J, Haratani K, Hayashi H (2018). Peripheral Blood Biomarkers Associated with Clinical Outcome in Non-Small Cell Lung Cancer Patients Treated with Nivolumab. J Thorac Oncol.

[11] Chu X, Zhao J, Zhou J (2020). Association of baseline peripheral-blood eosinophil count with immune checkpoint inhibitor-related pneumonitis and clinical outcomes in patients with non-small cell lung cancer receiving immune checkpoint inhibitors. Lung Cancer.

[12] Hirsch A, Ruffie P, Nebut M, Bignon J, Chretien J (1979). Pleural effusion: laboratory tests in 300 cases. Thorax.

[13] Light RW, Erozan YS, Ball WC (1973). Cells in pleural fluid. Their value in differential diagnosis. Arch Intern Med.

[14] Adelman M, Albelda SM, Gottlieb J, Haponik EF (1984). Diagnostic utility of pleural fluid eosinophilia. Am J Med.

[15] Krenke R, Nasilowski J, Korczynski P, Gorska K, Przybylowski T, Chazan R, Light RW (2009). Incidence and aetiology of eosinophilic pleural effusion. Eur Respir J.

[16] Clive AO, Kahan BC, Hooper CE (2014). Predicting survival in malignant pleural effusion: development and validation of the LENT prognostic score. Thorax.

[17] Anevlavis S, Kouliatsis G, Sotiriou I, Koukourakis MI, Archontogeorgis K, Karpathiou G, Giatromanolaki A, Froudarakis ME (2014). Prognostic factors in patients presenting with pleural effusion revealing malignancy. Respiration.

[18] Zamboni MM, Silva de CT, Baretta jr R, Cunha ET, Cardoso GP (2015). Important prognostic factors for survival in patients with malignant pleural effusion. BMC Pulm Med.

[19] Lee YS, Nam HS, Lim JH, Kim JS, Moon Y, Cho JH, Ryu JS, Kwak SM, Lee HL (2017). Prognostic impact of a new score using neutrophil-to-lymphocyte ratios in the serum and malignant pleural effusion in lung cancer patients. BMC Cancer.

[20] Fujii M, Yamashita T, Ishiguro R, Tashiro M, Kameyama K (2002). Significance of epidermal growth factor receptor and tumor associated tissue eosinophilia in the prognosis of patients with nasopharyngeal carcinoma. Auris Nasus Larynx.

[21] Ishibashi S, Ohashi Y, Suzuki T, Miyazaki S, Moriya T, Satomi S, Sasano H (2006). Tumor-associated tissue eosinophilia in human esophageal squamous cell carcinoma. Anticancer Res.

[22] Pretlow TP, Keith EF, Cryar AK, Bartolucci AA, Pitts AM, Pretlow TG, Kimball PM, Boohaker EA (1983). Eosinophil infiltration of human colonic carcinomas as a prognostic indicator. Cancer Res.

[23] Fernández-Aceñero MJ, Galindo-Gallego M, Sanz J, Aljama A (2000). Prognostic influence of tumor-associated eosinophilic infiltrate in colorectal carcinoma. Cancer.

[24] Takanami I, Takeuchi K, Gika M (2002). Immunohistochemical detection of eosinophilic infiltration in pulmonary adenocarcinoma. Anticancer Res.

[25] Costello R, O'Callaghan T, Sébahoun G (2005). Eosinophils and antitumour response. Rev Med Interne.

[26] Luna-Moré S, Florez P, Ayala A, Diaz F, Santos A (1997). Neutral and acid mucins and eosinophil and argyrophil crystalloids in carcinoma and atypical adenomatous hyperplasia of the prostate. Pathol Res Pract.

[27] Ono Y, Ozawa M, Tamura Y, Suzuki T, Suzuki K, Kurokawa K, Fukabori Y, Yamanaka H (2002). Tumor-associated tissue eosinophilia of penile cancer. Int J Urol.

[28] von Wasielewski R, Seth S, Franklin J, Fischer R, Hübner K, Hansmann ML, Diehl V, Georgii A (2000). Tissue eosinophilia correlates strongly with poor prognosis in nodular sclerosing Hodgkin’s disease, allowing for known prognostic factors. Blood.

[29] da Silva JM, Queiroz-Junior CM, Batista AC, Rachid MA, Teixeira MM, Silva da TA (2014). Eosinophil depletion protects mice from tongue squamous cell carcinoma induced by 4-nitroquinoline-1-oxide. Histol Histopathol.

[30] Reichman H, Karo-Atar D, Munitz A (2016). Emerging roles for eosinophils in the tumor microenvironment. Trends Cancer.

[31] Simon SCS, Utikal J, Umansky V (2019). Opposing roles of eosinophils in cancer. Cancer Immunol Immunother.

[32] Grisaru-Tal S, Itan M, Klion AD, Munitz A (2020). A new dawn for eosinophils in the tumour microenvironment. Nat Rev Cancer.

[33] Huland E, Huland H (1992). Tumor-associated eosinophilia in interleukin-2-treated patients: evidence of toxic eosinophil degranulation on bladder cancer cells. J Cancer Res Clin Oncol.

[34] Simon HU, Plötz S, Simon D, Seitzer U, Braathen LR, Menz G, Straumann A, Dummer R, Levi-Schaffer F (2003). Interleukin-2 primes eosinophil degranulation in hypereosinophilia and wells' syndrome. Eur J Immunol.

[35] Sosman JA, Bartemes K, Offord KP (1995). Evidence for eosinophil activation in cancer patients receiving recombinant interleukin-4: effects of interleukin-4 alone and following interleukin-2 administration. Clin Cancer Res.

[36] Bristol JA, Zhu M, Ji H, Mina M, Xie Y, Clarke L, Forry-Schaudies S, Ennist DL (2003). In vitro and in vivo activities of an oncolytic adenoviral vector designed to express GM-CSF. Mol Ther.

[37] Schaefer JT, Patterson JW, Deacon DH, Smolkin ME, Petroni GR, Jackson EM, Slingluff CL (2010). Dynamic changes in cellular infiltrates with repeated cutaneous vaccination: a histologic and immunophenotypic analysis. J Transl Med.

[38] Nakamura Y, Ozaki T, Yanagawa H, Yasuoka S, Ogura T (1990). Eosinophil colony-stimulating factor induced by administration of interleukin-2 into the pleural cavity of patients with malignant pleurisy. Am J Respir Cell Mol Biol.

[39] Carretero R, Sektioglu IM, Garbi N, Salgado OC, Beckhove P, Hämmerling GJ (2015). Eosinophils orchestrate cancer rejection by normalizing tumor vessels and enhancing infiltration of CD8(+) T cells. Nat Immunol.

[40] Salimi M, Wang R, Yao X (2018). Activated innate lymphoid cell populations accumulate in human tumour tissues. BMC Cancer.

[41] Ikutani M, Yanagibashi T, Ogasawara M (2012). Identification of innate IL-5-producing cells and their role in lung eosinophil regulation and antitumor immunity. J Immunol.

[42] Moral JA, Leung J, Rojas LA (2020). ILC2s amplify PD-1 blockade by activating tissue-specific cancer immunity. Nature.

[43] Heidecker J, Kaplan A, Sahn SA (2006). Pleural fluid and peripheral eosinophilia from hemothorax: hypothesis of the pathogenesis of EPE in hemothorax and pneumothorax. Am J Med Sci.

[44] Kwon BI, Hong S, Shin K, Choi EH, Hwang JJ, Lee SH (2013). Innate type 2 immunity is associated with eosinophilic pleural effusion in primary spontaneous pneumothorax. Am J Respir Crit Care Med.

[45] Tumino N, Martini S, Munari E (2019). Presence of innate lymphoid cells in pleural effusions of primary and metastatic tumors: Functional analysis and expression of PD-1 receptor. Int J Cancer.

